# Overexpression of P21-activated kinase 4 is associated with poor prognosis in non-small cell lung cancer and promotes migration and invasion

**DOI:** 10.1186/s13046-015-0165-2

**Published:** 2015-05-15

**Authors:** Songwang Cai, Zhiqiang Ye, Xiaohong Wang, Yuhang Pan, Yimin Weng, Sen Lao, Hongbo Wei, Lian Li

**Affiliations:** Department of Cardiothoracic Surgery, The Third Affiliated Hospital, Sun Yat-sen University, Guangzhou, Guangdong China; Department of Emergency, The Third Affiliated Hospital, Sun Yat-sen University, Guangzhou, Guangdong China; Department of Radiology, The Third Affiliated Hospital, Sun Yat-sen University, Guangzhou, Guangdong China; Department of Pathylogy, The Third Affiliated Hospital, Sun Yat-sen University, Guangzhou, Guangdong China; Department of Gastrointestinal Surgery, The Third Affiliated Hospital, Sun Yat-sen University, Guangzhou, Guangdong China; State Key Laboratory of Biocontrol, Sun Yat-sen (Zhongshan) University, Guangzhou, Guangdong China

**Keywords:** PAK4, LIM kinase 1, Non-small cell lung cancer, Migration, Invasion

## Abstract

**Background:**

P21-activated kinase 4 (PAK4), an effector of the Rho family protein Cdc42, is an important oncogene whose expression is increased in many human cancers and is generally positively correlated with advanced disease and decreased survival. However, little is known about the expression and biological function of PAK4 in human non-small cell lung cancer (NSCLC).

**Methods:**

PAK4 expression in NSCLC tissues and adjacent non-tumor tissues were assessed by immunohistochemistry, real-time PCR, and western blotting. Prognostic value of PAK4 expression was evaluated by Kaplan-Meier analysis and Cox regression. siRNA-mediated gene silencing and protein kinase assay was applied to demonstrate the role and the mechanism of PAK4 in lung cancer cell migration, invasion.

**Results:**

The results showed that PAK4 was overexpressed in NSCLC cell lines and human NSCLC tissues. PAK4 expression was detected both in the membranes and cytoplasm of NSCLC cancer cells in vivo. Moreover, increased expression of PAK4 was associated with metastasis, shorter overall survival, advanced stage of NSCLC. Furthermore, PAK4 expression was positively correlated with phosphorylation of LIMK1 expression levels. Knockdown of PAK4 in NSCLC cell lines led to reduce the phosphorylation of LIMK1, which resulted in decrease of the cell migration and invasion. In addition, PAK4 bound to LIMK1 directly and activated it via phosphorylation.

**Conclusions:**

These data demonstrate that PAK4 mediated LIMK1 phosphorylation regulates the migration and invasion in NSCLC. Therefore, PAK4 might be a significant prognostic marker and potential therapeutic molecular target in NSCLC.

## Background

Lung cancer is the leading cause of cancer-related death around the world, and approximately 80–85 % of lung cancers are non–small cell lung cancer (NSCLC) [1, 2], which accounts for up to 85 % of such deaths [3, 4]. The leading cause of death from NSCLC is metastasis, which occurs even before the diagnosis of lung cancer is made, leading to recurrence and treatment failure in patients [5–7]. Despite advances in therapeutic approaches, most patients are diagnosed at the advanced stages, and the 5 year survival rate remains less than 15 % [8]. Therefore, it is important to identify new predictive prognostic biomarkers and to better understand the mechanisms of disease progression.

P21-activated kinases (PAKs) are a family of serine/threonine (Thr) protein kinases positioned at the nexus of several oncogenic signaling pathways. Overexpression or mutational activation of PAK isoforms frequently occurs in various human tumors; given their involvement in cancer cell motility, survival, apoptosis, and metastasis, PAK isoforms are important regulators of cancer cell signaling networks [9]. Based on their amino acid sequences and their functions, 6 mammalian PAKs have been identified and classified into group I (PAK1, 2, 3) and group II (PAK4, 5, 6) PAKs [10, 11]. PAK4 was initially identified as an effector Cdc42, which is essential for regulating cytoskeleton reorganization and filopodia formation [12]. PAK4 is upregulated in many cancers and is an important oncogene that promotes proliferation [13–16] and migration [15–23], and suppresses apoptosis [24]. However, the role of PAK4 in NSCLC remains unclear.

In this study, we discovered that PAK4 is overexpressed in NSCLC and that its overexpression is associated with poor prognosis. Knockdown of PAK4 inhibited the migration and invasion of NSCLC cells. Furthermore, we found that PAK4 bound to LIM kinase 1 (LIMK1) directly and activated it via phosphorylation, which is required for tumor cell motility and invasion. These results suggest that PAK4 may play an important role in NSCLC cell migration and invasion, and is a potentially useful prognostic marker and therapeutic target.

## Materials and methods

### Tissue specimens and cell lines

NSCLC tissue samples and adjacent normal tissue samples were acquired following the obtainment of informed consent from the patients under institutional review board-approved protocols. NSCLC primary tissue samples of ten patients without metastasis and ten patients with metastasis and adjacent matched non-tumor tissues were collected between October 2012 and January 2013 at the Third Affiliated Hospital, Sun Yat-sen University (Guangzhou, China). The fresh tissue samples were immediately snap-frozen in liquid nitrogen. 210 NSCLC tissues were collected between January 2005 and January 2009. No patient had received treatment prior to enrolment in this study. All diagnoses were histopathologically confirmed. This study was approved by the institutional research ethics committee of The Third Affiliated Hospital, Sun Yat-sen University.

The following cell lines were used in this study: human bronchial epithelial cells (HBE) and NSCLC cells (A549, NCI-520, NCI-460, NCI-H596). All cell lines were obtained from Cell Bank, Chinese Academy of Sciences (Shanghai, China). The NSCLC cell lines were cultured in RPMI 1640 (Gibco, Invitrogen Life Technologies, Carlsbad, CA, USA) supplemented with 10 % newborn calf serum (Gibco, Invitrogen Life Technologies). HBE cells were maintained in keratinocyte serum-free medium with 25 μg/ml bovine pituitary extract and 0.2 ng/ml recombinant epidermal growth factor (Invitrogen Life Technologies). Cells were transfected with DNA constructs using siPORT™ NeoFX™ Transfection Agent (Ambion) for 5 min.

### Immunohistochemistry

Immunohistochemical staining was performed using a standard streptavidin-biotin-peroxidase complex method (EnVision™ Detection System; Dako, Copenhagen, Denmark). Tissue blocks were cut into 5-mm sections, deparaffinized with xylene, and rehydrated in a graded ethanol series. The sections were stained with anti-PAK4 (1:50; Cell Signaling Technology, Beverly, MA, USA) overnight at 4 °C.

The degree of immunostaining was reviewed and scored semiquantitatively by two independent observers. The staining index was calculated as the product of the proportion of positively stained tumor cells and the staining intensity. The former was scored as follows: 0 (0 % positive tumor cells), 1 (<10 %), 2 (10–35 %), 3 (35–70 %), and 4 (>70 %). Staining intensity was graded as follows: 0 (no staining), 1 (weak), 2 (moderate), and 3 (strong). The staining index was scored at 0–12. The cutoff values for high and low PAK4 expression were ascertained by measuring heterogeneity using log-rank testing with respect to overall survival. A staining index score of ≥6 indicated high PAK4 expression; a staining index score of <6 indicated low PAK4 expression.

### RNA isolation and quantitative real-time PCR

TRIzol reagent (Invitrogen Life Technologies) was used to extract total RNA. Complementary DNA (cDNA) was synthesized using a PrimeScript RT Reagent Kit (Promega, Madison, WI, USA). Real-time PCR was performed using Taqman universal PCR kit by an ABI 7900HT Fast Real-Time PCR system (Applied Biosystems, Foster City, CA, USA). The following primers were used: *PAK4* forward 5′-ATGTGGTGGAGATGTACAACAGCTA-3′ and reverse 5′-GTTCATCCTGGTGTGGGTGAC-3′; *U6* forward 5′-TGCGGGTGCTCGCTTCGGCAGC-3′ and reverse 5′-CCAGTGCAGGGTCCGAGGT-3′.

### Western blotting and immunoprecipitation

Western blotting was performed as described previously [25]. Proteins were separated using 10 % SDS-PAGE and then transferred to nitrocellulose membranes (Bio-Rad, Hercules, CA, USA). The membranes were incubated with rabbit anti-PAK4 (1:1000; Cell Signaling Technology), anti-LIMK1 (1:1000; Cell Signaling Technology), anti–p-LIMK1 (1:1000; Cell Signaling Technology), anti-cofilin (1:1000; Cell Signaling Technology), and anti–p-cofilin antibodies (1:1000; Cell Signaling Technology). The proteins were visualized using ECL reagents (Pierce, Rockford, IL, USA). Protein loading was estimated using rabbit anti-glyceraldehyde-3-phosphate dehydrogenase (GAPDH) antibody (Cell Signaling Technology). The intensity of protein fragments was quantified using GeneTools software (version 3.03; Syngene, Cambridge, UK). Three independent experiments were performed for all western blotting studies.

Immunoprecipitation assays were performed as described previously [25]. A549 or NCI-H520 cells (6 × 10^6^) were solubilized in 400 μl cell lysis buffer (1 % Triton X-100, 150 mM NaCl, 20 mM Tris-Cl [pH 7.4], 1 mM EDTA, 1 mM EGTA, 1 mM Na_3_VO_4_, 2.5 mM pyrophosphate, 1 mM glycerol phosphate, and a protease inhibitor mixture) for 10 min at 4 °C. The cell extract was immunoprecipitated with 4 μg PAK4 (Cell Signaling Technology) or LIMK1 (Cell Signaling Technology) antibody and then incubated with 60 μl Protein G Plus/Protein A-Agarose (Santa Cruz Biotech). The precipitated immunocomplexes were then boiled in Laemmli buffer and subjected to western blotting with anti-LIMK1 or anti-PAK4 antibody.

#### Immunofluorescence

Cells were cultured on cover glasses, fixed using paraformaldehyde, and permeabilized with 0.1 % Triton X-100 in TBS. The cover glasses were incubated with the primary antibodies (anti-PAK4, Cell Signaling Technology; anti-LIMK1, Cell Signaling Technology) at 1:50 dilutions. PAK4 was detected with an anti-goat secondary antibody conjugated to Alexa Fluor 488 (Invitrogen Life Technologies). LIMK1 was detected with an anti-rabbit secondary antibody conjugated to Alexa Fluor 555 (Invitrogen Life Technologies). The fluorescent staining was visualized using a 63× NA 1.3 oil objective on a confocal microscope (LSM 510 Meta; Carl Zeiss, Inc.). The co-localization was analyzed using Pearson’s correlation coefficient (full co-localization = 1.0) by the Image Pro Plus software.

### Protein kinase assay

PAK4 (WT) cDNA was cloned into a pET30a *Escherichia coli* expression vector. The mutant vectors PAK4 (S445N) and PAK4 (K350M) were constructed by site-directed mutagenesis. Recombinant activated PAK4 (S445N), kinase-defective PAK4 (K350M), and PAK4 (WT) proteins were purified from the *E. coli* expression systems. LIMK1 protein was purchased from Invitrogen Life Technologies. Equal amounts of the proteins were incubated in buffer containing 100 mM NaCl, 10 mM MgCl_2_, 50 mM HEPES (pH 7.5), 1 mM DTT, and 50 μM ATP for 30 min at 30 °C. The protein kinase assay reaction was terminated by the addition of 3× SDS sample buffer. Western blotting was used to detect LIMK1 phosphorylation at Thr508.

### Matrigel invasion assays and transwell migration assays

For Matrigel invasion assays, 5 × 10^4^ cells were added to a matrigel invasion chamber (BD Biosciences, CA, USA). FBS was added to the lower chamber as a chemoattractant. After 24 h, the non-invading cells were removed and invasive cells located on the lower side of the chamber were stained with crystal violet. Transwell migration assays were performed in a similar manner as the matrigel invasion assays but without matrigel on the filter.

### Statistical analysis

Data from three independent experiments are presented as the mean ± SE. All statistical analyses were performed using SPSS software (version 17.0; IBM, New York, NY, USA). Differences between variables were assessed using the *χ*^2^ test. For survival analysis, all patients with NSCLC were analyzed using Kaplan-Meier analysis. The differences in overall survival were analyzed using the log-rank test. Using the Cox regression model, multivariate survival analysis was performed on all parameters that were significant in the univariate analysis. *P-values* < 0.05 were considered significant.

## Results

### Pattern of PAK4 expression in human NSCLC cell lines or tissues

**Fig. 1:**
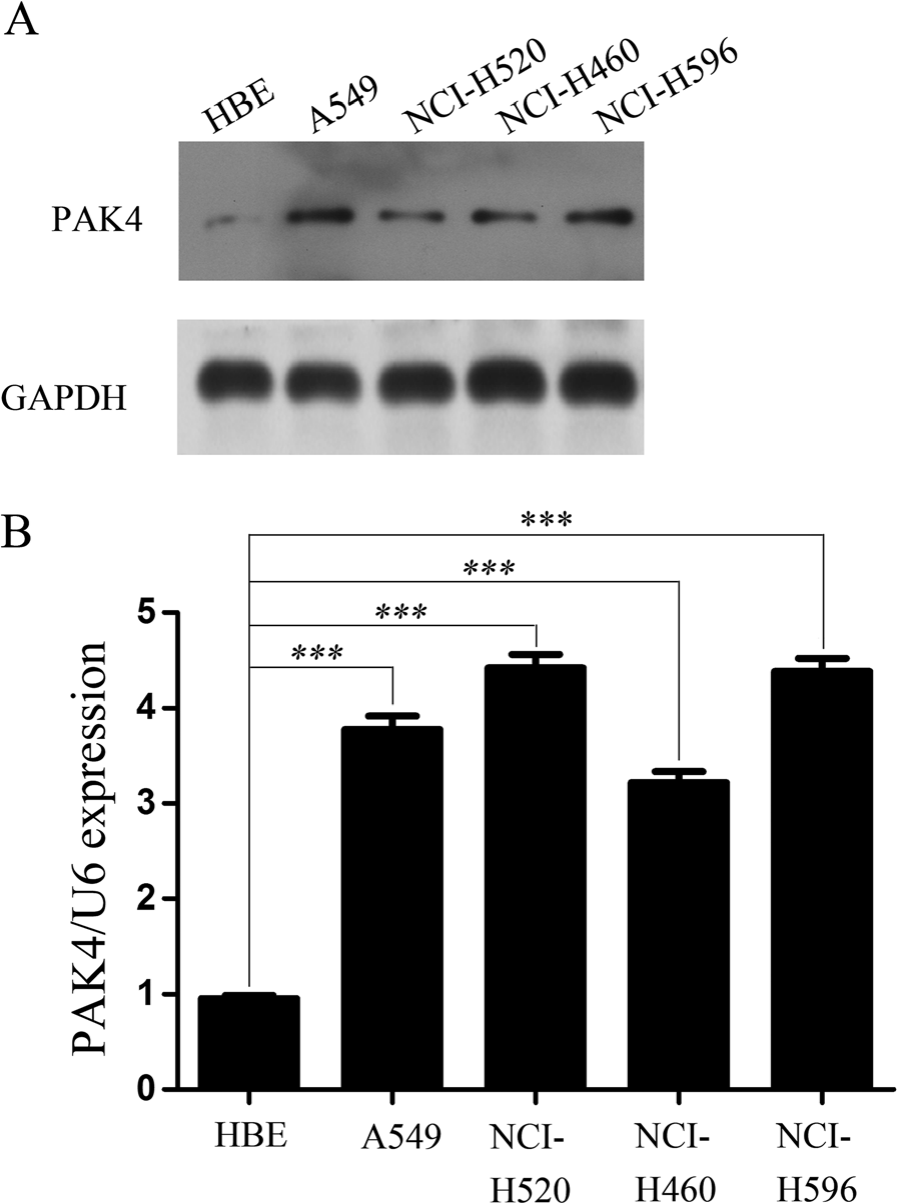
PAK4 expression in NSCLC cell lines. **a**: Western blots of PAK4 expression in HBE cell line and A549, NCI-520, NCI-460, and NCI-H596 NSCLC cell lines (*n* = 3 replicate experiments; *p* < 0.05 vs. HBE cells). **b**: Real-time PCR analysis of PAK4 expression in HBE, A549, NCI-520, NCI-460, and NCI-H596 cells (*n* = 3 replicate experiments; *p* < 0.001 vs. HBE cells)

PAK4 overexpression has been reported in many human cancers; however, its expression in NSCLC remains unclear. To explore PAK4 protein expression in NSCLC, we examined its expression in a human bronchial epithelial cell line (HBE) and several NSCLC cell lines using western blotting. All cancer cell lines expressed high levels of PAK4 protein compared with the HBE cell line (Fig. 1a). To determine whether PAK4 was overexpressed at the transcriptional level, we examined PAK4 mRNA levels in the HBE and NSCLC cell lines using real-time PCR. The levels of PAK4 mRNA in the NSCLC cell lines were significantly higher than that in the HBE cell line (Fig. 1b), indicating that PAK4 is overexpressed in NSCLC cell lines at both mRNA and protein levels compared to HBE cells.

**Fig. 2:**
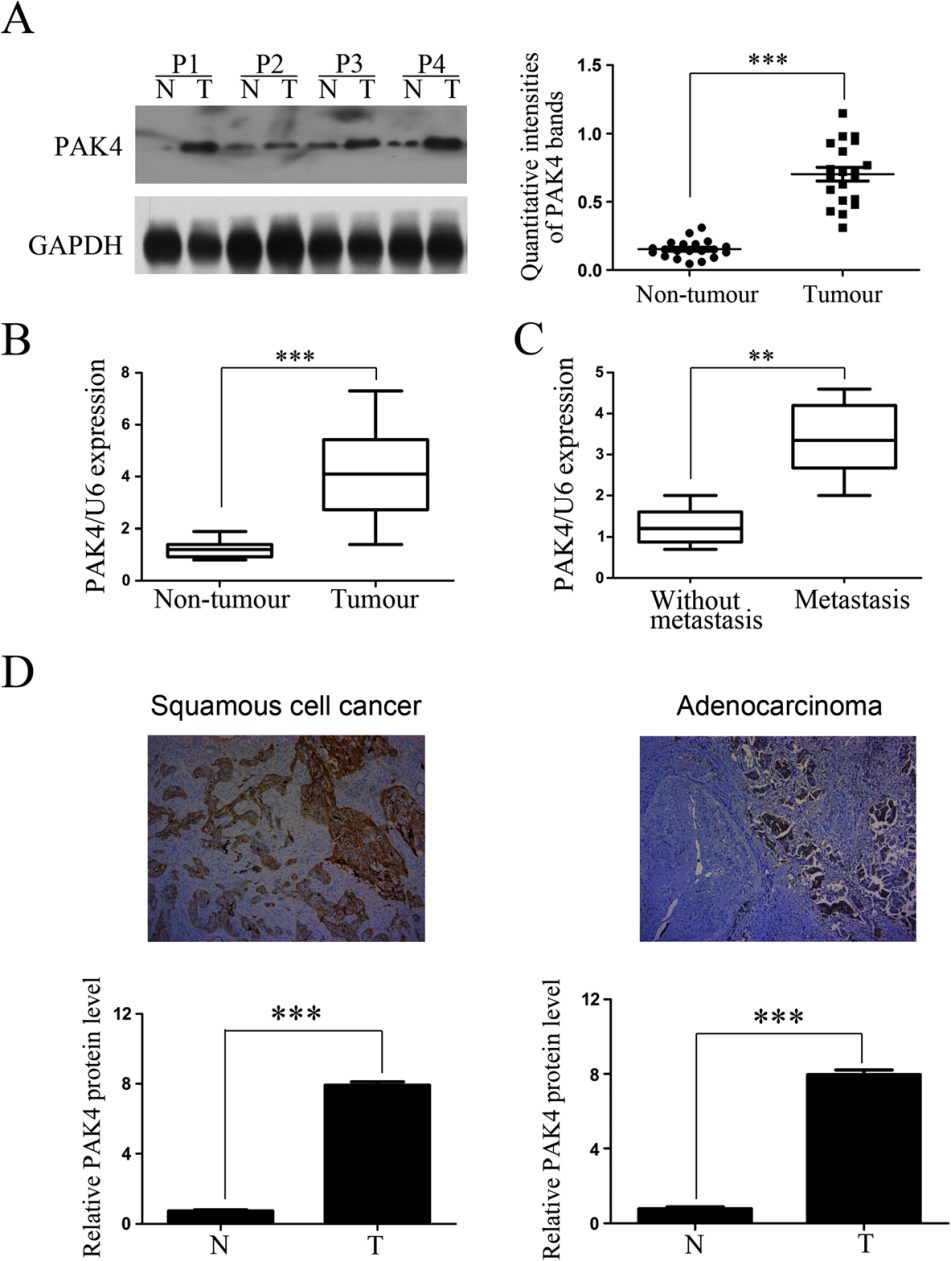
Expression of PAK4 and the association with progression in NSCLC. **a** Representative western blots of PAK4 expression (left) in NSCLC tissues (T) and matched adjacent non-tumor tissues (N); the quantitative intensities′ data of PAK4 protein bands of 20 NSCLC tissues and matched adjacent non-tumor tissues. **b**: The mRNA expression of PAK4 in 20 NSCLC tissues and matched adjacent non-tumor tissues by RT-PCR. **c**: PAK4 expression in primary NSCLC cancer tissues of 10 patients without metastasis and 10 patients with metastasis. **d**: Representative immunohistochemical staining (top panels) and statistical analysis (bottom panels) of squamous cell cancer (left) and adenocarcinoma (right) of human NSCLC tissues (T) and matched adjacent non-tumor tissues (N) under at × 40 original magnification. Positive cells are stained brown. P, patient

To investigate whether PAK4 was similarly upregulated in NSCLC tissues, western blotting and real-time PCR were performed on 20 NSCLC tissues (ten primary tissues without metastasis and ten primary tissues with metastasis) and matched adjacent non-tumor tissues. PAK4 was overexpressed at both protein and mRNA level in all 20 NSCLC tissues compared with the adjacent non-tumor tissues (Fig. 2a and b). Subgroup analysis showed that there was higher PAK4 mRNA in the metastatic NSCLC tissues compared to the primary NSCLC tissues (Fig. 2c).

### Association between PAK4 expression and NSCLC progression or prognosis

Based on the above results, we predicted that PAK4 overexpression would be associated with disease progression. To test this, 210 paired human NSCLC tissues and matched adjacent non-tumor tissues were stained using immunohistochemistry. Positive staining was observed in the NSCLC cell membranes and cytoplasm. The NSCLC tissues had significantly higher PAK4 staining scores than the adjacent non-tumor tissues (p < 0.01, Fig. 2d). There was stronger PAK4 staining, indicating higher expression, in 137 of 210 human NSCLC tissues (65.2 %). There were no significant differences among the 3 subtypes of NSCLC: adenocarcinoma, squamous cell carcinoma, and others (p = 0.454, Table 1). Moreover, higher PAK4 staining scores were positively correlated with differentiation, lymph node metastasis, distant metastasis, and clinical stage. These results reveal that PAK4 overexpression was associated with NSCLC progression.

**Table 1:** Correlation of PAK4 expression with clinicopathological variables in 210 cases of NSCLC

Variables	PAK4			P Value*
	All cases	Low expression	High expression	
	(n = 210)	(n = 73)	(n = 137)	
Age (years)				
≤60	103	35	68	0.816
>60	107	38	69	
Gender				
Male	136	48	88	0.826
Female	74	25	49	
Histological type				
SqCC	66	19	47	0.454
A	124	46	78	
Other	20	8	12	
Differentiation				
Low	115	32	83	0.020
Moderate + high	95	41	54	
T factor				
T1 + T2	112	40	72	0.757
T3 + T4	98	33	65	
Lymph node				
N0 + N1	97	41	56	0.034
N2 + N3	113	32	81	
Distant metastasis				
M0	176	67	109	0.022
M1	34	6	28	
Clinical stage				
I + II	81	35	46	0.042
III + IV	129	38	91	

*χ ^2^ test; *SqCC* squamous cell cancer; A

**Fig. 3:**
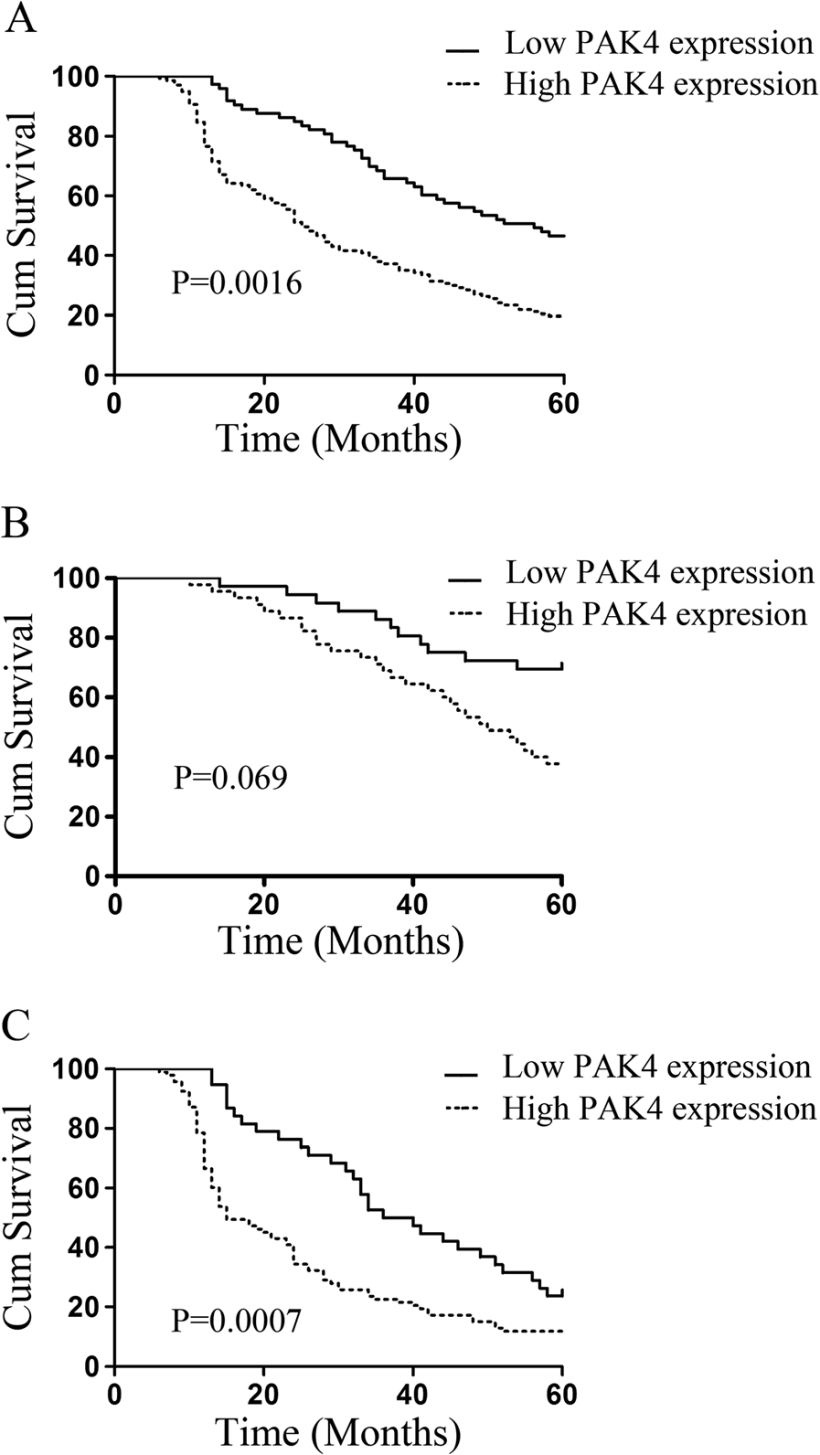
Kaplan-Meier survival curve illustrating the prognostic significance of PAK4 expression in NSCLC. **a**: The patients (n = 210) were divided into high or low PAK4 expression groups according to the proportion of positively stained tumor cells and staining intensity. 5 year survival was calculated using the Kaplan-Meier method and analyzed using the log-rank test. **b**: 5 year survival was calculated only in the stage I + II cancer patients. **c**: 5 year survival was calculated only in the stage III + IV cancer patients

Kaplan-Meier analysis and log-rank testing showed that overall survival was significantly different between patients with upregulated PAK4 and patients with downregulated PAK4 (p = 0.0016, Fig. 3a); patients with upregulated PAK4 had shorter overall survival. Subgroup analysis also indicated that patients with high PAK4 expression had poor overall survival in both early stage (I + II) and later stage (III + IV) patients (Fig. 3b, c). Moreover, differentiation, lymph node invasion, distant metastasis, and clinical stage correlated with survival. When the clinicopathological variables that were significant in univariate analysis were adopted as covariates, multivariate Cox regression analysis indicated that overexpression of PAK4 protein was an independent prognostic factor for poor survival (p < 0.01, Table 2). These results suggested that overexpression of PAK4 was positively associated with NSCLC progression and might serve as an independent predictor of poor survival.

**Table 2:** Univariate and multivariate analysis of factors associated with overall survival of patients with NSCLC

Clinical variable	Case number	HR (95 % CI)	*P Value*
Uinvariate analysis			
PAK4 (High vs. Low)	137/73	1.627(1.195–2.215)	0.002
Age (>60 vs. ≤60)	107/103	1.084(0.812–1.446)	0.584
Gender (Male vs. Female)	136/74	1.165(0.864–1.571)	0.317
Histological type (SqCC vs A vs. Other)	66/124/20	1.069(0.832–1.374)	0.602
Distant metastasis (M1 vs. M0)	34/176	5.259(3.512–7.875)	0.000
T factor (T3 + T4 vs. T1 + T2)	98/112	1.090(0.817–1.455)	0.557
Lymph node (N2 + N3 vs. N0 + N1)	113/97	5.349(3.852–7.426)	0.000
Clinical stage (III + IV vs. I + II)	129/81	7.266(5.106–10.339)	0.000
Differentiation (Moderate + high vs. Low)	95/115	1.892(1.412–2.536)	0.000
Multivariate analysis			
PAK4 (How vs. Ligh)	137/73	0.483(0347–0.672)	0.000
Differentiation (Moderate + high vs. Low)	95/115	1.191(0.853–1.663)	0.305
Distant metastasis (M1 vs. M0)	34/176	3.703(2.389–5.740)	0.000
Lymph node (N2 + N3 vs. N0 + N1)	113/97	1.311(0.725–2.372)	0.371
Clinical stage (III + IV vs. I + II)	129/81	5.nn3.069–11.080)	0.000

### Influence of PAK4 knockdown on NSCLC cell migration and invasion

**Fig. 4:**
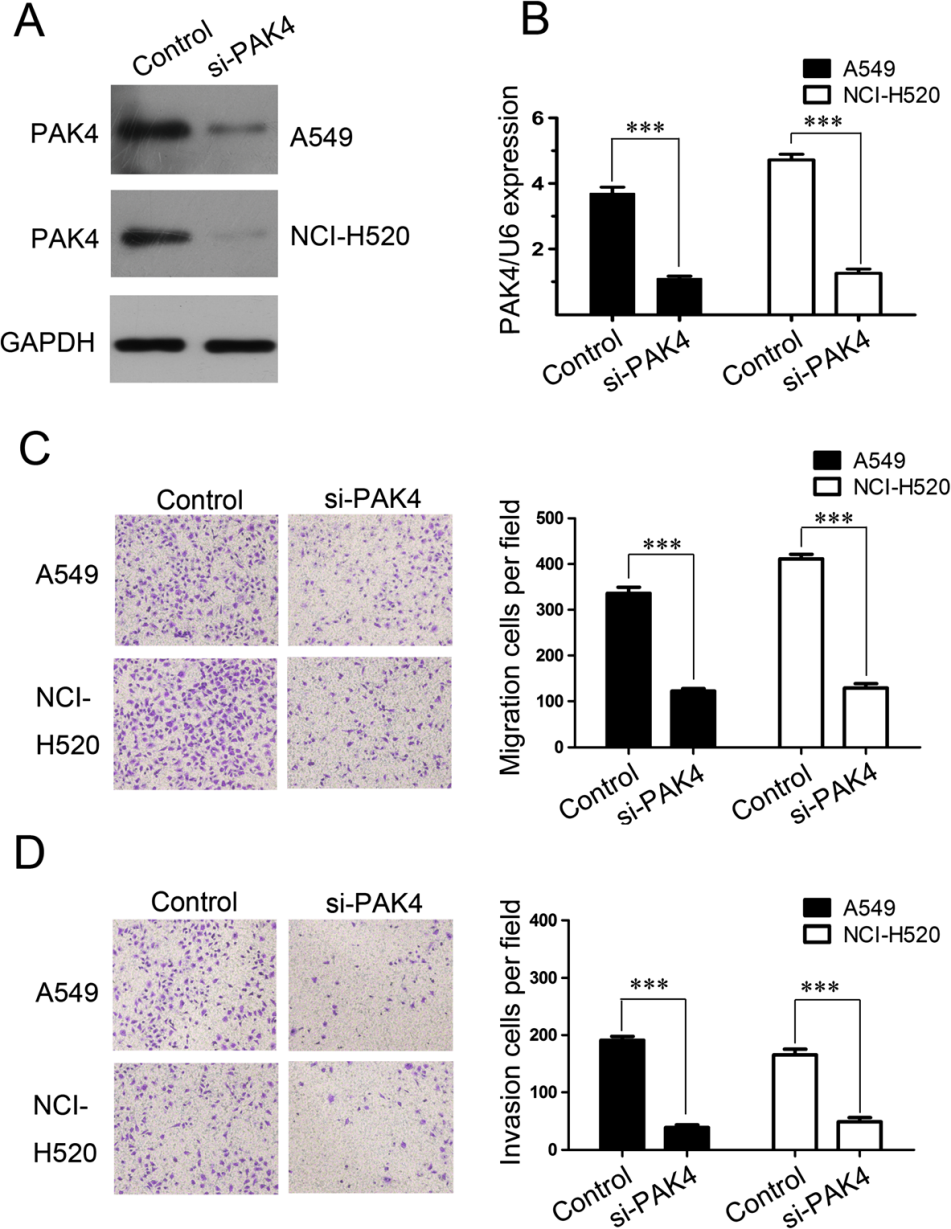
PAK4 knockdown suppressed NSCLC cell migration and invasion. The protein (**a**) and mRNA (**b**) expression of PAK4 in A549 and NCI-H520 cells transfected with siRNA(si-PAK4) or control siRNA(control) . Transwell migration assay (**c**) and Matrigel invasion assay (**d**) of A549 and NCI-H520 cells transfected with si-PAK4 or control under at × 40 original magnification

These above data prompted us to further examine the mechanism that PAK4 mediated in the progression of NSCLC. First, PAK4 expression was downregulated by siRNA-mediated gene silencing. Knockdown of endogenous PAK4 in the A549 and NCI-H520 cells was confirmed using western blotting and real-time PCR (Fig. 4a and b). Then, Transwell migration assay and Matrigel invasion assay were carried out to explore the potential biological function of PAK4 in NSCLC. PAK4 knockdown suppressed A549 and NCI-H520 cell migration compared with the controls (Fig. 4c), and dramatically reduced A549 and NCI-H520 cell invasiveness (Fig. 4d). Transfection with si-PAK4 decreased the numbers of migrated and invasive A549 and NCI-H520 cells by >3-fold as compared with cells transfected the control (both, p < 0.01). These results indicate that PAK4 knockdown suppresses NSCLC cell migration and invasion.

### The role of LIMK1 for PAK4-mediated NSCLC cell migration and invasion

**Fig. 5:**
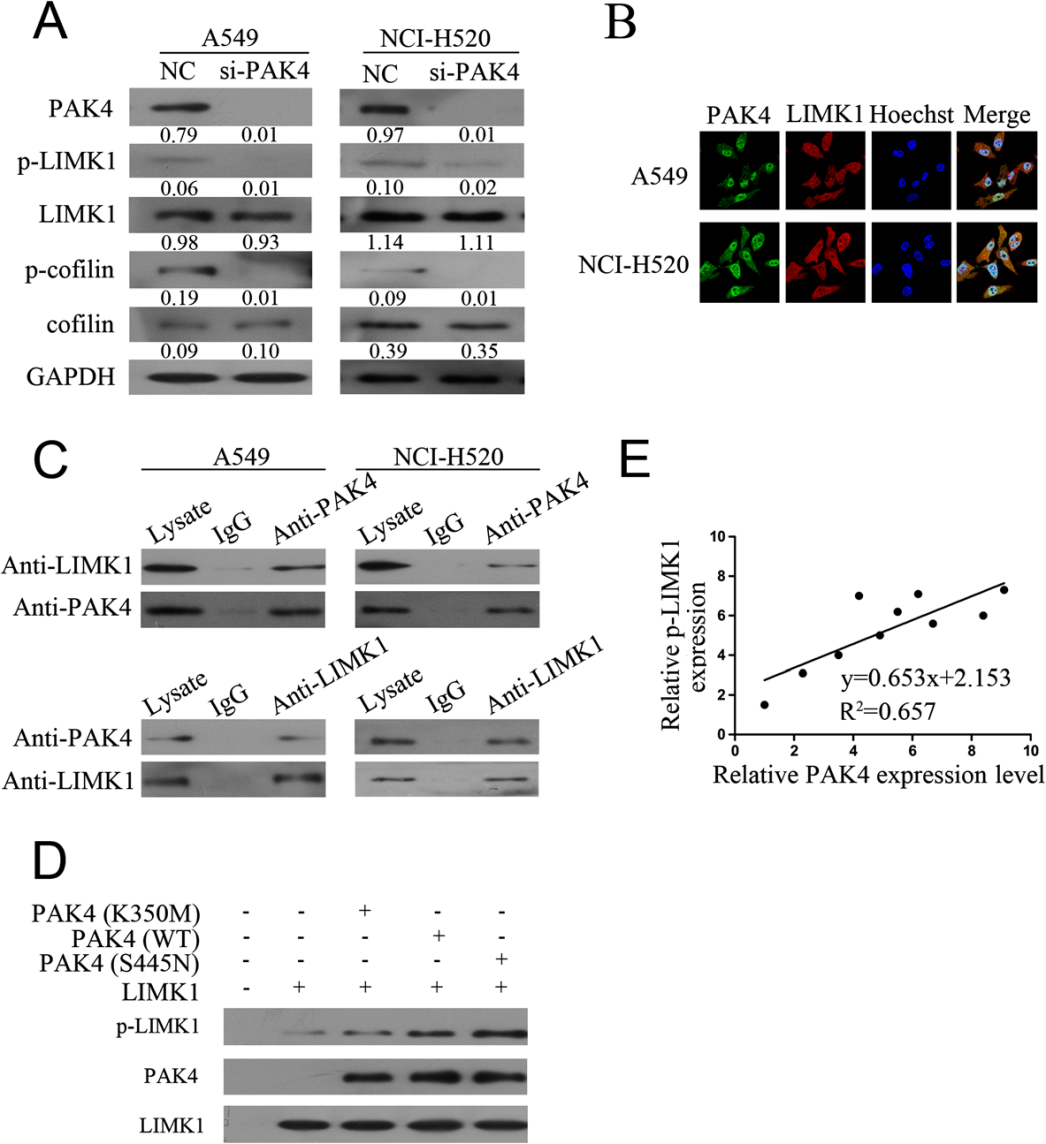
LIMK1 was required for PAK4-mediated NSCLC cell migration and invasion. **a**: The protein expression of LIMK1, p-LIMK1, cofilin, and p-cofilin in A549 and NCI-H520 cells transfected with si-PAK4 or control by western blotting. **b**: Immunofluorescent staining for PAK4 (green) and LIMK1 (red) in A549 cells (upper panels) or in NCI-H520 cells (lower panels). Nuclei were stained with Hoechst33258 (blue). **c**: A549 and NCI-H520 cell lysates were immunoprecipitated with PAK4 antibody (top panels) or LIMK1 antibody (bottom panels) and subjected to western blotting to ascertain LIMK1 and PAK4 interaction. **d**: In vitro kinase assay using purified activated PAK4 (S445N), kinase-defective PAK4 (K350M), PAK4 (WT), and LIMK1 protein. The amount of p-LIMK1, PAK4, and LIMK1 were measured using western blotting. **e**: Correlations between the protein level of PAK4 and the p-LIMK1 in human NSCLC tissues (*n* = 10). NC, negative control; IgG, immunoglobulin G

Cell migration requires reorganistion of the actin cytoskeleton. PAK4, an effector of Cdc42, elicits their response via interaction with downstream effector proteins. Previous studies showed that LIMK and its downstream cofillin activated by PAK4 plays an important role in promoting actin polymerization and defining the direction of cell motility [26]. We examined whether LIMK1 and cofilin are involved in regulating NSCLC cell line migration and invasion. Following siRNA knockdown of PAK4, LIMK1, cofilin, and their respective phosphorylation were examined using western blotting. PAK4 knockdown significantly reduced the phosphorylation of LIMK1 and cofilin, whereas the total expression levels of LIMK1 and cofilin did not change (Fig. 5a). These results indicate that PAK4 might regulate the the phosphorylation of LIMK1 and cofilin in NSCLC cell.

To explore the possibility that PAK4 phosphorylated LIMK1 in vivo, we examined whether PAK4 physically interacted with LIMK1 in the A549 and NCI-H520 cells using by immunofluorescent staining. The results showed that PAK4 (green) and LIMK1 (red) were predominantly localized within the cytoplasm (Fig. 5b). The degree of co-localization was determined using Pearson’s correlation coefficient; the mean ± SE of PAK4 and LIMK1 colocalization was 0.72 ± 0.02 (n = 30) in the A549 cells and 0.79 ± 0.03 (n = 30) in the NCI-H520 cells. These results showed that PAK4 and LIMK1 generally co-localized in the PC-3 and DU145 cells.

To investigate the role of PAK4 in the process of LIMK1 and cofilin phosphorylation, co-immunoprecipitation assays were performed. Cell lysates were incubated with PAK4 antibody, the immunocomplex was purified, separated by SDS-PAGE, and immunoblotted with a LIMK1 antibody. The LIMK1 protein was present in the complex immunoprecipitated with the anti-PAK4 antibody (Fig. 5c, top); PAK4 was also present in the reciprocal immunoprecipitation with the anti-LIMK1 antibody (Fig. 5c, bottom). In addition, neither PAK4 nor LIMK1 were detected in the immunocomplex in association with the control immunoglobulin G, indicating the specificity of the observed co-association. These results show that PAK4 interacts specifically with LIMK1 in NSCLC cells.

PAK4 is an important oncogene in many cancers, and many of its functions are dependent on PAK4 kinase activity. To determine whether PAK4 directly phosphorylated LIMK1, we performed an in vitro kinase assay using purified wild-type PAK4 (WT) and LIMK1 protein, and found that LIMK1 was phosphorylated by PAK4 (Fig. 5d). We also used purified PAK4 with different activities, activated PAK4 (S445N), kinase-defective PAK4 (K350M), and LIMK1 protein in the in vitro kinase assay. LIMK1 phosphorylation in the presence of PAK4 (K350M) was significantly lower than that in the presence of PAK4 (WT) or PAK4 (S445N) (Fig. 5d). These data indicate that PAK4 could phosphorylate LIMK1 protein.

To further reveal the relation between PAK4 and phosphorylation of LIMK1 in clinical NSCLC tissues, we examined the expression of PAK4 and p-LIMK1 in 20 human NSCLC tissues using western blotting. The extent of PAK4 upregulation was positively correlated with the amount of p-LIMK1 (R^2^ = 0.657, p < 0.05) (Fig. 5e), suggesting that the effect of PAK4 kinase activity on LIMK1 is clinically relevant in human NSCLC tissues.

**Fig. 6:**
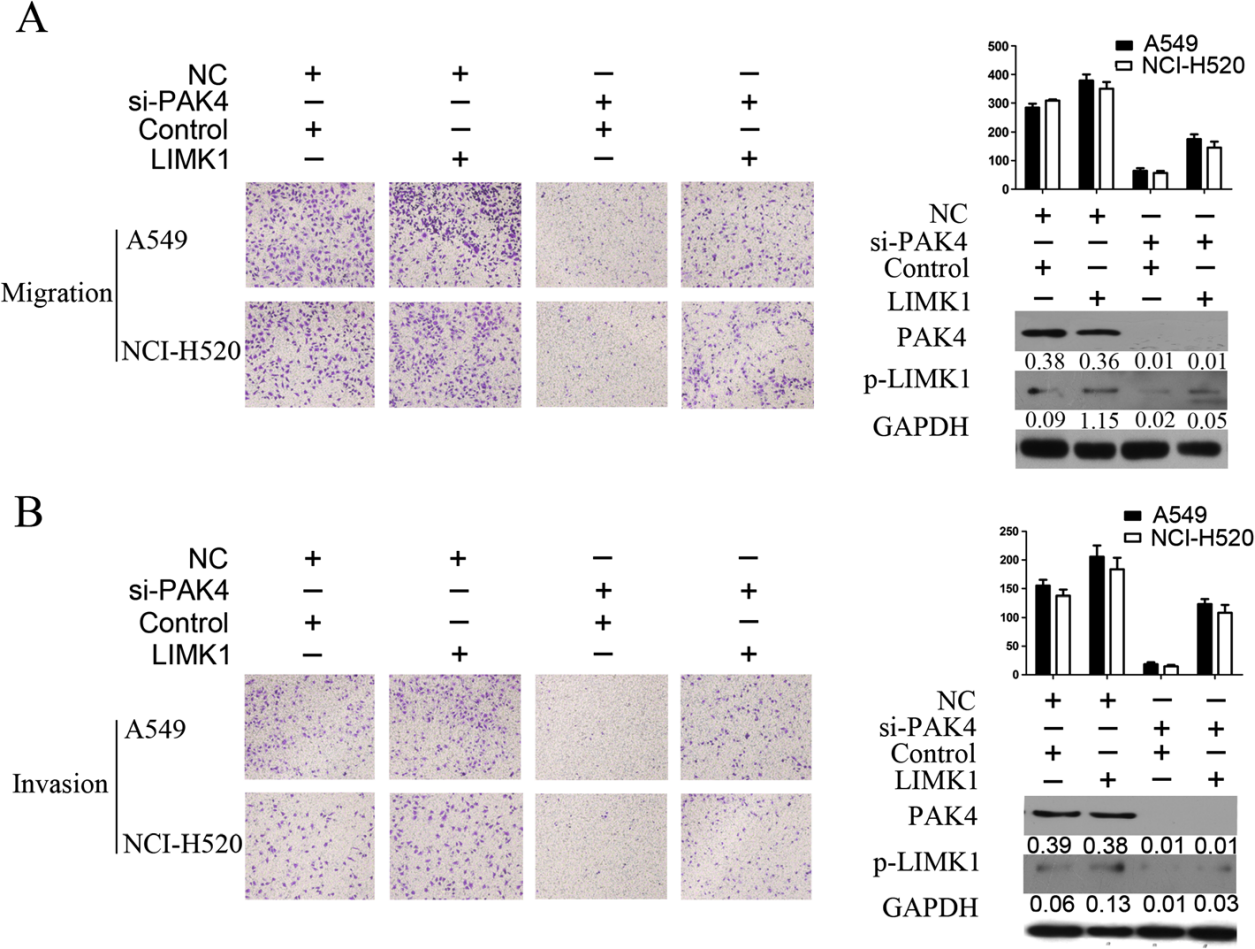
LIMK1 overexpression rescued the effects of si-PAK4 on A549 and NCI-H520 cell migration and invasion. A549 or NCI-H520 cells were transiently transfected with Si-PAK4 or LIMK1 or both, and then seeded for migration (**a**) and invasion assay (**b**) under at × 40 original magnification. NC, negative control

To reveal whether LIMK1 was involved in the PAK4-mediated NSCLC cell migration and invasion, we co-transfected A549 and NCI-H520 cells with si-PAK4 or LIMK1 plasmid. LIMK1 rescued the effects of PAK4 knockdown on A549 and NCI-H520 cell migration and invasion (Fig. 6). These above results suggest that LIMK1 phosphorylation is required for PAK4-mediated NSCLC cell migration and invasion.

## Discussion

Although PAK4 is an important oncogene in many cancers, the role of PAK4 in NSCLC remains obscure. In this study, we detected PAK4 overexpression in NSCLC cell lines and human NSCLC tissues, and found that PAK4 overexpression was correlated significantly with clinical stage, differentiation, lymph node metastasis, and distant metastasis. More importantly, upregulated expression of PAK4 in NSCLC patients was associated with shorter overall survival. These results show that PAK4 is an important prognostic marker and potential therapeutic target in NSCLC.

Upregulated PAK4 promotes cell migration in several cancers [13–23]. However, the role of PAK4 in NSCLC cell migration remains unclear. As far as we know, this is the first study to reveal that PAK4 increase NSCLC cell migration and invasion. Furthermore, PAK4 regulation of cell migration is dependent on the downstream pathway of membrane-type 1 matrix metalloproteinase (MT1-MMP) in choriocarcinoma [15, 16], c-Src/mitogen-activated protein kinase kinase 1 (MEK1)/extracellular signal-regulated kinase (ERK)1/2 and MMP-2 in ovarian cancer [16], LIMK1/cofilin in prostate cancer [17], MMP-2 in glioma [19], and superior cervical ganglia 10 (SCG10) or LIMK1/cofilin in gastric cancer [20, 21]. We revealed that PAK4 promoted migration by directly binding to LIMK1 and activating it via phosphorylation. It has been reported that the PAK4-LIMK1-cofilin signaling pathway promotes cell migration in prostate cancer and gastric cancer [17, 20]. Other signal pathways through which PAK4 mediates NSCLC migration require further exploration.

LIMK1, a downstream effector of PAK4, is an important regulator of cytoskeletal organization involved in cell migration [27, 28]. LIMK1 is overexpressed in lung cancer and is associated with high tumor-nodes-metastasis (TNM) stage and lymph node metastasis [29]. Our experiments also showed that LIMK1 was overexpressed in NSCLC tissues, especially in tissues with metastasis (data not shown). Furthermore, si-LIMK1 suppressed the migration and invasion of lung cancer cells [29]. However, how modulating LIMK1 promotes lung cancer cell migration remains unclear. In this study, we found that LIMK1 interacted directly with PAK4 and acted as a substrate to promote cell migration and invasion in NSCLC. Furthermore, overexpression of LIMK1 rescued the effects of PAK4 knockdown on NSCLC cell migration and invasion. These findings show that PAK4 increased NSCLC cell migration by phosphorylating LIMK1.

CDK5 kinase regulatory subunit-associated protein 3 (CDK5RAP3) in hepatocellular cancer [22] and hepatocyte growth factor (HGF) and follicle-stimulating hormone (FSH) in ovarian cancer [16] activate PAK4 to promote cell migration. HGF and c-Met are overexpressed in NSCLC [30]. However, the upstream pathways of PAK4 in NSCLC cell migration remain unclear. We intend to explore the mechanism of PAK4 activation in NSCLC in future studies.

## Conclusion

In the present study, increased PAK4 expression was associated with differentiation, lymph node metastasis, distant metastasis, clinical stage, and an unfavorable prognosis in patients with NSCLC. Our findings suggest that thePAK4-LIMK1 pathway may be related to the progression of NSCLC and that PAK4 may be significant prognostic marker for this disease.

## References

[1] Ferlay J, Shin HR, Bray F, Forman D, Mathers C, Parkin DM (2010). Estimates of worldwide burden of cancer in 2008: GLOBOCAN 2008. Int J Cancer.

[2] Qu Y, Wu X, Yin Y, Yang Y, Ma D, Li H (2014). Antitumor activity of selective MEK1/2 inhibitor AZD6244 in combination with PI3K/mTOR inhibitor BEZ235 in gefitinib-resistant NSCLC xenograft models. J Exp Clin Cancer Res.

[3] Tsuboi M, Ohira T, Saji H, Miyajima K, Kajiwara N, Uchida O (2007). The present status of postoperative adjuvant chemotherapy for completely resected non-small cell lung cancer. Ann Thorac Cardiovasc Surg.

[4] Wang J, Shen Q, Shi Q, Yu B, Wang X, Cheng K (2014). Detection of ALK protein expression in lung squamous cell carcinomas by immunohistochemistry. J Exp Clin Cancer Res.

[5] Lin G, Sun L, Wang R, Guo Y, Xie C (2014). Overexpression of muscarinic receptor 3 promotes metastasis and predicts poor prognosis in non-small-cell lung cancer. J Thorac Oncol.

[6] Chen X, Guan X, Zhang H, Xie X, Wang H, Long J (2015). DAL-1 attenuates epithelial-to mesenchymal transition in lung cancer. J Exp Clin Cancer Res.

[7] Chen Y, Liu H, Wu W, Li Y, Li J (2013). Osteopontin genetic variants are associated with overall survival in advanced non-small-cell lung cancer patients and bone metastasis. J Exp Clin Cancer Res.

[8] Jemal A, Siegel R, Ward E, Hao Y, Xu J, Thun MJ (2009). Cancer statistics, 2009. CA Cancer J Clin.

[9] Radu M, Semenova G, Kosoff R, Chernoff J (2014). PAK signalling during the development and progression of cancer. Nat Rev Cancer.

[10] Kumar R, Gururaj AE, Barnes CJ (2006). p21-activated kinases in cancer. Nat Rev Cancer.

[11] Fryer BH, Field J (2005). Rho, Rac, Pak and angiogenesis: old roles and newly identified responsibilities in endothelial cells. Cancer Lett.

[12] Abo A, Qu J, Cammarano MS, Dan C, Fritsch A, Baud V (1998). PAK4, a novel effector for Cdc42Hs, is implicated in the reorganization of the actin cytoskeleton and in the formation of filopodia. EMBO J.

[13] Callow MG, Clairvoyant F, Zhu S, Schryver B, Whyte DB, Bischoff JR (2002). Requirement for PAK4 in the anchorage-independent growth of human cancer cell lines. J Biol Chem.

[14] Tabusa H, Brooks T, Massey AJ (2013). Knockdown of PAK4 or PAK1 inhibits the proliferation of mutant KRAS colon cancer cells independently of RAF/MEK/ERK and PI3K/AKT signaling. Mol Cancer Res.

[15] Zhang HJ, Siu MK, Yeung MC, Jiang LL, Mak VC, Ngan HY (2011). Overexpressed PAK4 promotes proliferation, migration and invasion of choriocarcinoma. Carcinogenesis.

[16] Siu MK, Chan HY, Kong DS, Wong ES, Wong OG, Ngan HY (2010). p21-activated kinase 4 regulates ovarian cancer cell proliferation, migration, and invasion and contributes to poor prognosis in patients. Proc Natl Acad Sci U S A.

[17] Ahmed T, Shea K, Masters JR, Jones GE, Wells CM (2008). A PAK4-LIMK1 pathway drives prostate cancer cell migration downstream of HGF. Cell Signal.

[18] Li Z, Zhang H, Lundin L, Thullberg M, Liu Y, Wang Y (2010). p21-activated kinase 4 phosphorylation of integrin beta5 Ser-759 and Ser-762 regulates cell migration. J Biol Chem.

[19] Kesanakurti D, Chetty C, Rajasekhar Maddirela D, Gujrati M, Rao JS (2012). Functional cooperativity by direct interaction between PAK4 and MMP-2 in the regulation of anoikis resistance, migration and invasion in glioma. Cell Death Dis.

[20] Li X, Ke Q, Li Y, Liu F, Zhu G, Li F (2010). DGCR6L, a novel PAK4 interaction protein, regulates PAK4-mediated migration of human gastric cancer cell via LIMK1. Int J Biochem Cell Biol.

[21] Guo Q, Su N, Zhang J, Li X, Miao Z, Wang G (2014). PAK4 kinase-mediated SCG10 phosphorylation involved in gastric cancer metastasis. Oncogene.

[22] Mak GW, Chan MM, Leong VY, Lee JM, Yau TO, Ng IO (2011). Overexpression of a novel activator of PAK4, the CDK5 kinase-associated protein CDK5RAP3, promotes hepatocellular carcinoma metastasis. Cancer Res.

[23] Lu W, Xia YH, Qu JJ, He YY, Li BL, Lu C (2013). p21-activated kinase 4 regulation of endometrial cancer cell migration and invasion involves the ERK1/2 pathway mediated MMP-2 secretion. Neoplasma.

[24] Li X, Minden A (2005). PAK4 functions in tumor necrosis factor (TNF) alpha-induced survival pathways by facilitating TRADD binding to the TNF receptor. J Biol Chem.

[25] Zhu B, Li X, Zhang Y, Ye C, Wang Y, Cai S (2013). Cross-talk of alpha tocopherol-associated protein and JNK controls the oxidative stress-induced apoptosis in prostate cancer cells. Int J Cancer.

[26] Kidera Y, Tsubaki M, Yamazoe Y, Shoji K, Nakamura H, Ogaki M (2010). Reduction of lung metastasis, cell invasion, and adhesion in mouse melanoma by statin-induced blockade of the Rho/Rho-associated coiled-coil-containing protein kinase pathway. J Exp Clin Cancer Res.

[27] McConnell BV, Koto K, Gutierrez-Hartmann A (2011). Nuclear and cytoplasmic LIMK1 enhances human breast cancer progression. Mol Cancer.

[28] Dan C, Kelly A, Bernard O, Minden A (2001). Cytoskeletal changes regulated by the PAK4 serine/threonine kinase are mediated by LIM kinase 1 and cofilin. J Biol Chem.

[29] Chen Q, Jiao D, Hu H, Song J, Yan J, Wu L (2013). Downregulation of LIMK1 level inhibits migration of lung cancer cells and enhances sensitivity to chemotherapy drugs. Oncol Res.

[30] Gumustekin M, Kargi A, Bulut G, Gozukizil A, Ulukus C, Oztop I (2012). HGF/c-Met overexpressions, but not met mutation, correlates with progression of non-small cell lung cancer. Pathol Oncol Res.

